# Supplementary material for: Risk factors for neonatal hypothermia at Arba Minch General Hospital, Ethiopia
Source: PLoS One. 2022 Dec 22;17(12):e0267868. doi: 10.1371/journal.pone.0267868 (PMC9778974; doi:10.1371/journal.pone.0267868)
Supplement: Attachment — Submitted filename: comment on hypotermia.docx [file pone.0267868.s003.docx]

**Comments**

Thank you for invitation to review Neonatal Hypothermia and Its Associated Risk Factors among Newborns at Arba Minch General Hospital, Ethiopia. The article is presented in a structured way and is well written. Below I provide some remarks on the manuscript parts. Additionally, I suggest the authors to discuss the implication of the result and include the ethical approval number.

1. In the abstract section, there is no convincing gap that shows why the research is needed?
2. In the abstract section, you should clearly indicate the design
3. I appreciate the authors use of multi-state Markov model to characterize the outcome variable.
4. In the introduction section, you need to show the previous works, how your finding adds to the existing knowledge, and gap of your work. Furthermore, you need to show the impact of the problem using current evidence. E.g., neonatal mortality due to hypothermia in Ethiopia
5. On the method part, it is too shallow and it didn’t address all important components of research methodology. Clearly indicate the design? You didn’t mention quality control measures. You need to show how you measure your outcome variable in detail.
6. On the result and discussion parts, I recommend the author to write pertinent findings only? I appreciate the authors trying different measurement of outcome variable. The discussion is well written and structured. However, it lacks the implication of the results. Try to use current evidences.
7. The paper needs language edition.
