# Supplementary material for: Risk factors for neonatal hypothermia at Arba Minch General Hospital, Ethiopia
Source: PLoS One. 2022 Dec 22;17(12):e0267868. doi: 10.1371/journal.pone.0267868 (PMC9778974; doi:10.1371/journal.pone.0267868)
Supplement: Attachment — Submitted filename: Response to Reviewers.docx [file pone.0267868.s005.docx]

Review Manuscript PONE-D-22-10601 – 29.8.22

Authors’ Response to Reviewers’ Comments
**Title:** **Risk Factors for Neonatal Hypothermia at Arba Minch General Hospital, Ethiopia**

Authors: **Tegenu Tento, Tilahun Asena, Meseret Alemayehu and Asmare Wube.**

First of all, authors would like to convey our sincere thanks and gratitude to the respected reviewers for their valuable comments and suggestions for the improvement of the technical quality of the manuscript. The authors have tried their level best to incorporate all the comments of the respected reviewers. We strongly hope that the quality of the revised manuscript has been improved enormously after taking the respected reviewers’ comments and suggestions into consideration.

**Reviewer Comments:**

**Reviewer 1**

The point-wise authors’ response to the reviewers’ comments are given in the following:

# Recommendation to the Editor

Requires expert statistical review

- Response: expertise revie is made as suggested by the reviewers
  General comment

Neonatal hypothermia is an important public health issue strongly associated with neonatal death – the subject is therefore of importance. Little longitudinal data is available, which makes this work of interest.

The text needs in depth English editing including grammar and spell-checking to be understandable.

- Response: English editing including grammar and spelling-checked as suggested by the reviewers

The raw data is not well presented.

- Corrected as the review’s suggestions

Does not add substantially new medical information, although proposes a quite novel method to possibly reach such conclusions, based on very short term longitudinal data.s

The Markov model stipulates that a future state depends only on prior state – for temperature not the case (multifactorial)

# Specific comments

# Title

Shorten: Risk Factors for Neonatal Hypothermia at Arba Minch General Hospital, Ethiopia

- Response: corrected as suggested by the reviewers

# Abstract

English editing needed: review phrasings.

- Response: corrected as suggested by the reviewers

Method: ‘However’ should be replaced by why 391 were included only. The Appearance Pulse Grimace Activity Respiration should be written Apgar score in Methods. The Apgar score has been developed by Virginia Apgar in the 50-ties and the mnemonic ‘Appearance Pulse Grimace Activity Respiration’ is not recognisable by many as the proper score.

- Response: corrected as suggested by the reviewers

Results: Expressing Markov model decimal fractions is an unusual way to express probabilities in the medical literature. Unclear whether ‘progressing to normal’ state means improvement from severe to moderate and from moderate to mild or only to normal state (>36.5°C). Selection of HR cut-off for the 3 states unclear.

- Response: corrected as suggested by the reviewers

Conclusions: Confirms what is well known: VLBW, low Apgar and high HR associated with longer time of recovery.

As data probably from NICU only, inference can be done on NICU care only: ‘even’ should be cancelled the …’even in the NICU’.

- Response: corrected as suggested by the reviewers

# Background

Needs English editing, particularly the last paragraph.

- Response: English editing is done as suggested by the reviewers

Definition of low body temperature required: eg: ‘core body temperature’.

The metabolic rate raises well before the reduction of the core body temperature, that is during cold stress whilst core body temperature is still normal – correct.

Whilst ‘cold stress’ is indeed defined by WHO as reported, in physiologic terms there is a difference between cold stress and mild hypothermia. Please add ‘according to WHO’.

‘…and should receive skilled care as soon as possible’ gives the impression this is only necessary for severe hypothermia, but skilled care is needed already in ‘physiologic cold stress’ to prevent hypothermia.

- Response: corrected as suggested

Change ‘control ‘with ‘maintain’, as newborns can control, but loose much more temperature due to the body configuration, and it is more the vaso-motor and metabolic capacity that is overrun the ‘control’.

- Corrected as the review’s suggestions

Correct the 17 million hypothermia deaths – this is impossible. It is the number of newborns suspected to become hypothermic every year around 2010 (from the cited review paper). If possible, find a more recent reference.

- Response: corrected and further references are added

The message of the sentence ‘Because newborn health…’ cannot be understood. Please modify.

- Response: modified as suggested

As details are given immediately after the last sentence in Methods, the time frame may be omitted for improved reading.

- Response: modified as suggested

Methods

## General

English editing required.

- Response: English editing is done as suggested by the reviewers

It remains unknown what the general interventions were in hospital for thermal care.

The exclusion of babies that did not recover within 3 hours or died in hypothermia was supposedly necessary for the Markov model work but is a major limitation. That needs at least a detailed result (specification of how many) and a discussion of the limitation of the model.

- For this research purposes the infants died within 3 hours are excluded because of the objective of the study and as exclusion criteria set for the study purpose.

## The data

What is ‘secondary data’? is it just retrospective data?

- Response: modified as suggested

What means ‘on a standard time basis’?

Up to 7 temperature recordings every 30 minutes, starting from admission? If not, please specify.

- Response: modified as suggested

The Hospital is mentioned in full too many times. It is sufficient to reporte it once in the methods – avoid repetitions that make reading cumbersome.

- Response: modified as suggested

In the Inclusion and exclusion paragraph: The lowest state (worsening to severe hypothermia) may have been defined with some assumptions to use the Markov model as a combined outcome (severe hypothermia and/or death and/or not recovered within 3 hours) to avoid exclusion of the most pertinent outcomes for clinicians.

In the Variables paragraph: The choice to combine severe and moderate hypothermia appears to be based on model requirements more than clinical arguments. However, separating mild hypothermia (cold stress) from normal is a good choice. As stated above, by including other severe outcomes closely related to temperature (death and non-recovery) in the severe group may have sufficiently increased the number to make the Markov model work.

- Response: modified as suggested

In the Data analysis method: Reference should be referenced in ‘Vancouver’ style and do not require the year in brackets, but the reference number. The detailed mathematical resolution of the Markov model is probably more that of a methodological paper, but in my view acceptable as the model is not common in the medical literature.

- Response: modified as suggested

As a clinician, even more so without the raw data, I am not confident enough to confirm the application of the Markov model and its additional statistical analyses that need an expert statistician review.

# Ethical considerations

Although there is an ethical consideration in the full-text MS, there is no ethics statement in the MS summary although human participants are evaluated.

Although described as such, a wording including ‘anonymously’ is needed.

- Suppletory file is included and for further reference readers are suggested too red over there

# Results

English editing and spell-checking required

- Response: English editing and spelling-checked as suggested by the reviewers

Give only percentage of males or females as they add up to 100%.

- Response: modified as suggested

Obstetric complications, RDS and perinatal asphyxia need a definition in the Methods.

Give only percentage of preterms or terms as they add up to 100%.

- Response: modified as suggested

Tables need to be referenced in the text, such as ‘Table X summarizes transitions from one state to another within the …’.

- Response: corrected as suggested by the reviewers

First paragraph: It is essential to understand the data, how many of the excluded were persistently hypothermic for more than 3 hours and how many died. Table 1 may easily be omitted

- Response: modified as suggested

3.1: Table 2 gives clearer information than the text paragraph that can be practically omitted.

- Response: corrected as suggested by the reviewers

3.2: The first paragraph (Transition intensities…) is more an explanatory text for table 3 and not suitable to start a new results section.

- Response: corrected as suggested by the reviewers

The paragraph should probably start with saying that these are the results of the model for covariates. Concerning ‘The effect was 0.4086911…’, first, in the text it is not clear what this number means (better understandable in the table) and second, more than two or three decimals after the point are clinically unreasonable even if mathematically correct. The paragraph could be significantly shortened and improved by focusing on the 3 significant changes and giving a more clinical appreciation such as (I am not sure from the given test/numbers that my interpretation is indeed correct): Birth weight influenced by 41% the chance of improvement from sever/moderate to mild hypothermia.

- Response: corrected as suggested by the reviewers

3.3: Within the examined timeframe of 180 minutes… The text can be shortened as data is more clearly visible from the table.

- Response: modified as suggested

3.4: This should be better explained in the text: Table 5 does not add any additional understanding.

- Response: modified as suggested

3.5: To use the wording ‘Estimated survival time…’ is confusing, as according to the methods all deceased newborns have been excluded beforehand. This statistical wording should be rephrased accordingly.

- Response: modified as suggested

I do not understand what the authors want to tell us with the last paragraph and table 6. The null hypothesis needs at least to be mentioned. The last sentence is a hypothesis that may be at best fit in the discussion.

- Response: corrected as suggested by the reviewers

# Figures and Tables

## Figures

Figure 1: Detail somewhere on this graph exclusions.

- Response: modified as suggested

Figure 2: Misleading figure title as all newborns survived. Rephrase to make it clear. I understand that the ‘survival plot’ concerns ‘remaining’ in a certain state (survival) and that a ‘lower survival’ is better as there is a change into a better state, that is remaining less time in hypothermia. The last sentence of the first paragraph, however, contradicts this. Please improve wording for better understanding and check if this last sentence is correct. My interpretation is that newborns in state 2 (mild hypothermia) remain less time in mild hypothermia, that those in state 1, that remain longer in severe/moderate hypothermia.

- Response: corrected as suggested by the reviewers

## Tables

Table 1: Needs to be referenced in the Results text. Avoid duplication! Results either in table or in text, not both. This table can easily be omitted. Mean birth weight may be an interesting parameter to add to this table or better to the text paragraph.

- Response: corrected as suggested by the reviewers

Table 2: In the table text, the time frame to/from is a useful information to add. Otherwise, the reader does not easily understand it is in hours (and not days, or weeks...).

Sever = Severe and moderate

Indicate what (…) means

- Response: modified as suggested

Table 3: It would be nice to have the raw data too in this table. I cannot see from the table how the covariates were used and neither does the text give enough specification. I suppose that for instance for weight, normal weight was compared to LBW; this should be specified for each co-variable though. ‘Sever’ should read ‘severe or moderate’. Table text needs to include that it shows the 95%CI. Reduce number of decimals. The star (*) indicates significant changes and should be added to the table text. This table gives a little bit the impression of ‘fishing for significance’.

- Response: modified as suggested

Table 4: Why is this table not reporting values for mild hypothermia too? Mild hypothermia is a severe condition for the neonate where metabolic defence mechanisms have been exhausted. Presenting only severe/moderate hypothermia unreasonably minimizes the effect of mild hypothermia. Please add these data.

- Response: modified as suggested

Table 5: to be omitted and to be detailed in text.

- Response: modified as suggested

Table 6: Two tables that do not really fit together. Probably best to describe the two test results in the text.

- Response: modified as suggested

# Discussion

Globally the discussion is poor and hard to understand.

- Response: corrected as suggested by the reviewers

English editing necessary

- Response: English editing is done as suggested by the reviewers

It is strange that not one single normal temperature baby changed to mild hypothermia during the first 3 hours. If this is not an artefact, this means that once normal, no hypothermia ever occurred again (although this was not followed up > 3h). This finding merits a discussion point.

- Response: corrected as suggested by the reviewers

There would be some space to discuss whether the difference in time to improvement (e.g., 33 vs 48 min) is not only statistically but also clinically relevant.

- Response: modified as suggested

**The text**

I don’t really understand the first sentence.

The second paragraph comments an 0.18 chance (18%?) of transitioning from state 2 (mild) to state 1 (moderate/severe), but table 2 reports 0.05. What is right?

Also, this is a transition to a worse state, contrary to what just follows (English formulation problem?). The references 8,9 and 19 follow a statement of present data and do not make sense here. A limitation of the work is no doubt, that the number of severe hypothermia was very small and ‘artificially’ reduced by excluding death. Indeed, <32°C core temperature, the likelihood of death is extremely high (Demtse et al. Hypothermia in Preterm Newborns: Impact on Survival. Glob Pediatr Health. 2020 doi: 10.1177/2333794X20957655).

- Response: modified as suggested

Although the first 3 hours may play a significant role in thermoregulation this cannot be deducted from the present work that excluded newborns over 3 hours and those that died or remained hypothermic thereafter.

- Response: modified as suggested

The discussion on **weight** does not add new information. Low birthweight is associated with more hypothermia and more difficulties to correct it. Other differences between newborns (Apgar score) may influence correction too, but heartrate may more reflect the metabolic state with increased oxygen and substrate consumption already for newborns in cold stress and clearly in any state of hypothermia. The paragraph needs rewording.

- Response: corrected as suggested by the reviewers

The discussion on **APGAR** is confusing and does not make clear what is present results and what comes from the literature. It is not astonishing though that newborns with low APGAR score may have more hypothermia and may be more difficult to bring into normothermia. Once again, the metabolic and respiratory contribution to a complicated adaptational process (low APGAR) will compete for these resources that will be required for thermal adaptation. The paragraph need rewording.

- Response: corrected as suggested by the reviewers

The discussion on **pulse rate** is too broad. It should be stated whether it is high or low pulse rate that was associated with worsening of the thermal state. The reader should not have to go back to the results to finally find out that the present study showed a higher pulse rate associated with hypothermia, whereas here other authors are cited that report a decreased pulse rate is the most common symptom of hypothermia [27]. However, reference 27 cites term newborns with encephalopathy in therapeutic hypothermia, possibly not comparable.

- Response: modified as suggested

# Conclusion

The conclusion is too vague and broad. The past tense should be used for reference to the present results. English review is required.

- Response: English editing is done as suggested by the reviewers

Unclear whether high or low birthweight increased the risk (of improvement ?) from severe/moderate to mild hypothermia. The rest of this first paragraph would much better fit in the introduction as there is no direct relation to the present work.

- Response: modified as suggested

The paragraph on pulse rate needs rephrasing. ‘re-beating’ is unclear. High pulse rate should be used instead of faster beating. This is in contrast with reference 27 cited before.

- Response: modified as suggested

# Recommendations

I would not call this section ‘recommendations’ but add them to the Discussion.

The first paragraph is general knowledge and should be omitted

- Response: modified as suggested

The second paragraph has its rights in that that present data pinpoints processes for improvement.

A third section could specify future research and I would suggest focussing on deceased and persistent hypothermia, the sickest newborns that were excluded here.

- Response: corrected as suggested by the reviewers

**My personal conclusions** of the results:

1. Exclusion of persistent hypothermic and deceased new-borns limits these findings to the less sick new-borns.
2. For hypothermic survivors within the first 3 hours of life, VLBW, low APGAR and high pulse rate had the strongest association with hypothermia and required the longest time to improvement/recovery.

# References

Ok

**Reviewer Comments:**

**Reviewer 2**

The point-wise authors’ response to the reviewers’ comments are given in the following:

**Comments**

Thank you for invitation to review Neonatal Hypothermia and Its Associated Risk Factors among Newborns at Arba Minch General Hospital, Ethiopia. The article is presented in a structured way and is well written. Below I provide some remarks on the manuscript parts. Additionally, I suggest the authors to discuss the implication of the result and include the ethical approval number.

1. In the abstract section, there is no convincing gap that shows why the research is needed?

Response: corrected as suggested by the reviewers

2. In the abstract section, you should clearly indicate the design

Response: corrected as suggested by the reviewers

3. I appreciate the authors use of multi-state Markov model to characterize the outcome variable.

Response: thanks

4. In the introduction section, you need to show the previous works, how your finding adds to the existing knowledge, and gap of your work. Furthermore, you need to show the impact of the problem using current evidence. E.g., neonatal mortality due to hypothermia in Ethiopia

Response: corrected as suggested by the reviewers

5. On the method part, it is too shallow and it didn’t address all important components of research methodology. Clearly indicate the design? You didn’t mention quality control measures. You need to show how you measure your outcome variable in detail.

Response: correction is made as suggested by the reviewers

6. On the result and discussion parts, I recommend the author to write pertinent findings only? I appreciate the authors trying different measurement of outcome variable. The discussion is well written and structured. However, it lacks the implication of the results. Try to use current evidences.

Response: corrected as suggested by the reviewers

7. The paper needs language edition.

Response: English editing is done as suggested by the reviewers
