# Supplementary material for: Risk factors for neonatal hypothermia at Arba Minch General Hospital, Ethiopia
Source: PLoS One. 2022 Dec 22;17(12):e0267868. doi: 10.1371/journal.pone.0267868 (PMC9778974; doi:10.1371/journal.pone.0267868)
Supplement: Attachment — Submitted filename: Response to Reviewers.docx [file pone.0267868.s006.docx]

**Response to Reviewers**

Cover Letter

Dear Editor,

We appreciate you and the reviewers for your precious time in reviewing our paper and providing valuable comments. It was your valuable and insightful comments that led to possible improvements in the current version. The authors have carefully considered the comments and tried our best to address every one of them. We hope the manuscript after careful revisions meet your high standards. The authors welcome further constructive Comments if any. Below we provide the point-by-point responses. All modifications in the manuscript have been highlighted.

Sincerely,

Tilahun Ferede, PhD

Feredetilahun14@gmail.com
Assistant Professor

Department of Statistics

Arba Minch University

**Response to Reviewer**

1. If the authors have adequately addressed your comments raised in a previous round of review and you feel that this manuscript is now acceptable for publication, you may indicate that here to bypass the “Comments to the Author” section, enter your conflict of interest statement in the “Confidential to Editor” section, and submit your "Accept" recommendation.

Reviewer #1: (No Response)

**Response: Thanks for your comment. We revised the entire manuscript for comments to the author and revised all comments accordingly.**

2. Is the manuscript technically sounds, and do the data support the conclusions?
The manuscript must describe a technically sound piece of scientific research with data that supports the conclusions. Experiments must have been conducted rigorously, with appropriate controls, replication, and sample sizes. The conclusions must be drawn appropriately based on the data presented.

Reviewer #1: Yes

Response: Thank you for your comments. We have gone through your comments carefully
and tried our best to address them one by one. We hope the manuscript has been improved accordingly.

3. Has the statistical analysis been performed appropriately and rigorously?

Reviewer #1: Yes

Response: Thank you for your comments and responses.

4. Have the authors made all data underlying the findings in their manuscript fully available?

The [PLOS Data policy](http://www.plosone.org/static/policies.action#sharing) requires authors to make all data underlying the findings described in their manuscript fully available without restriction, with rare exception (please refer to the Data Availability Statement in the manuscript PDF file). The data should be provided as part of the manuscript or its supporting information, or deposited to a public repository. For example, in addition to summary statistics, the data points behind means, medians and variance measures should be available. If there are restrictions on publicly sharing data—e.g. participant privacy or use of data from a third party—those must be specified.

Reviewer #1: Yes

Response: Thank you very much for the comment.

5. Is the manuscript presented in an intelligible fashion and written in Standard English?

PLOS ONE does not copyedit accepted manuscripts, so the language in submitted articles must be clear, correct, and unambiguous. Any typographical or grammatical errors should be corrected at revision, so please note any specific errors here.

Reviewer #1: No

Response: Thank you very much for the comment. We did our best to correct these mistakes

6. Review Comments to the Author

Please use the space provided to explain your answers to the questions above. You may also include additional comments for the author, including concerns about dual publication, research ethics, or publication ethics. (Please upload your review as an attachment if it exceeds 20,000 characters)

Reviewer #1: Thank you for your effort in addressing the comments. The majority of the comments were addressed. However, the authors should critically consider revising the typo errors. Even you should be wise when writing words. Look at your response to author’s cover letter “We sincerely hope and believe that the revised manuscript will be acceptable for publication in your esteemed journal, BMC journal”.

6. Review Comments to the Author

Please use the space provided to explain your answers to the questions above. You may also include additional comments for the author, including concerns about dual publication, research ethics, or publication ethics. (Please upload your review as an attachment if it exceeds 20,000 characters)

Reviewer #1: Thank you for your effort in addressing the comments. The majority of the comments were addressed. However, the authors should critically consider revising the typo errors. Even you should be wise when writing words. Look at your response to author’s cover letter “We sincerely hope and believe that the revised manuscript will be acceptable for publication in your esteemed journal, BMC journal”.

PLOS authors have the option to publish the peer review history of their article ([what does this mean?](https://journals.plos.org/plosone/s/editorial-and-peer-review-process#loc-peer-review-history)). If published, this will include your full peer review and any attached files.

If you choose “no”, your identity will remain anonymous but your review may still be made public.

**Do you want your identity to be public for this peer review?** For information about this choice, including consent withdrawal, please see our [Privacy Policy](https://www.plos.org/privacy-policy).

Reviewer #1: **Yes:** Addis Eyeberu

Response: Thank you very much for the comment.
